# Measuring relational security in forensic mental health services

**DOI:** 10.1192/pb.bp.116.055509

**Published:** 2017-12

**Authors:** Verity Chester, Regi T. Alexander, Wendy Morgan

**Affiliations:** 1 Department of Psychiatry, Partnerships in Care Learning Disability Services; 2 Leicestershire Partnership NHS Trust; 3 Department of Psychology, Social Work and Allied Health Sciences, Glasgow Caledonian University

## Abstract

**Aims and method** Relational security is an important component of care and risk assessment in mental health services, but the utility of available measures remains under-researched. This study analysed the psychometric properties of two relational security tools, the See Think Act (STA) scale and the Relational Security Explorer (RSE).

**Results** The STA scale had good internal consistency and could highlight differences between occupational groups, whereas the RSE did not perform well as a psychometric measure.

**Clinical implications** The measures provide unique and complimentary perspectives on the quality of relational security within secure services, but have some limitations. Use of the RSE should be restricted to its intended purpose; to guide team discussions about relational security, and services should refrain from collecting and aggregating this data. Until further research validates their use, relational security measurement should be multidimensional and form part of a wider process of service quality assessment.

Serious incident investigations in forensic mental health services have been linked to relational security breakdowns.^1^ However, relational security has been described as the ‘poor relation’^2^ when compared with physical or procedural security. This is most likely due to definition difficulties. Chester & Morgan^3^ noted that numerous conceptualisations of relational security exist, all referencing different phenomena, such as ‘therapeutic relationships’ and ‘boundaries’, without describing how such concepts can affect security.^3^ The authors noted that the most practically useful definitions emphasise exactly how relational issues affect security, in two stages: (1) staff knowledge of patients and therapeutic relationships; and (2) how patient knowledge and therapeutic relationships are used to foresee potential and manage actual security incidents, for example: ‘The professional relationships between staff and patients so that … the staff to get to know … their patients, their moods and problems, to facilitate interventions before these become major problems, or lead to incidents of a security nature’ (p. 171).^4^ As such, relational security is intrinsically linked to short- and long-term assessment and management of risk in mental health and forensic services.

Relational security is therefore a service quality indicator of interest within forensic and general mental health services. Its measurement should allow practitioners to explore background causes and respond accordingly with appropriate interventions. Due to the definitional complexities, there are subsequent challenges to measurement. Previously available tools only measure partial aspects of relational security or individual patient needs.^3^ However, two tools have been developed in recent years, the Relational Security Explorer^5^ and the See Think Act scale.^2^ As limited research has examined the clinical utility of these measures, the present study examines their psychometric properties to support the aim of relational security measurement.

## Method

### Design

The study employed a within-participants (reliability testing) and between-participants (assessing the measures' ability to identify differences between occupational groups according to the variables level of security, ward gender and length of experience working in secure services) cross-sectional questionnaire design.

### Participants

Participants were recruited from a forensic service for people with intellectual disability. As guidance recommends that relational security implementation should involve all occupational groups working within secure services,^6^ the study invited all staff who have contact with patients as part of their role to take part in the study (*n* = 216), and of these 41% (*n* = 89) responded. The majority of the participants were female (63%), and the average length of service working in secure services was 6 years (range 0.12–20). The majority of participants were from the nursing department (57%), followed by occupational therapy (16%), psychology (9%), social work (6%), psychiatry (2%) and housekeeping (8%), and two participants identified their occupation as ‘other’. The level of security in which respondents worked in at the time of the study was medium (23.6%), low (27%) and locked rehabilitation (16.9%). There are no national data available which describe the demographics of the forensic mental health workforce, although the reported characteristics reflected the socio-demographic and occupational breakdown of the study service and the broader mental health workforce as a whole,^7^ being predominantly female and with nursing staff comprising the largest department followed by other members of the multidisciplinary team. As participants responded anonymously, it was not possible to undertake any analyses comparing participants with non-responders.

### Measures

#### See Think Act (STA) scale^2^

The STA scale is the first attempt to create a direct measure of relational security. The 28 items were developed from the Department of Health conceptualisation of relational security,^6^ and reflect relational security scenarios (e.g. ‘We speak up if we think we can see that a colleague has been put in a difficult position that could weaken security’). Individual staff members complete the questionnaire in relation to the ward they work on, and select how closely their ward team resembles provided statements on a 4-point Likert scale, with scores of 3 ‘Just like our team’, 2 ‘Quite like our team’, 1 ‘A little like our team’ and 0 ‘Not like our team’. A principal components analysis confirmed a four-component structure of the measure: Therapeutic Risk Management, Pro-Social Team Culture, Boundaries and Patient Focus.^2^ Initial examinations indicated good convergent validity with related measures (e.g. EssenCES^8^), and internally consistent subscales.^2^ There is currently no normative data available for the STA scale.

#### The Relational Security Explorer (RSE)^5^

The RSE is a tool designed to help clinical teams working within secure settings to communicate and assess their competence in relational security. The tool requires users to provide a numerical score of their team's confidence in eight areas of relational security: Therapy, Boundaries, Patient Mix, Patient Dynamic, Personal World, Physical Environment, Visitors and Outward Connections on a scale ranging from 1 (no confidence) to 10 (extremely confident). The RSE was not developed as a psychometric measure; however, the tool requests clinical teams to provide a numerical score of their confidence in each of the eight areas. In doing so, the tool lends itself for use as an outcomes measure and, anecdotally, the authors are aware of service's collecting and analysing this data, despite it not being validated for this purpose. This is in line with the suggestion that when numerical indices and cut-off points are available, clinical decisions tend to be reduced to those numbers.^9^ Participants were asked to complete the RSE on an individual, rather than a team basis, to investigate the tool performance as an outcomes measure.

### Procedure

The researcher arranged one-to-one meetings with staff eligible for the research, at which informed consent was sought. Once obtained, staff members were asked to complete and return the questionnaires. A debrief form was provided for participants, which detailed further information about the study.

### Ethics

Ethical approval was obtained from the London Metropolitan University Research Ethics Review Panel. The National Research Ethics Service Committee for the East of England – Norfolk was also approached for ethical review of the project, who advised that as the relational security measures were being used in routine clinical practice within the study service, the study does not require National Health Service ethical approval.^10,11^

### Data analysis

Prior to analysis, assumption testing for parametric tests was completed. The assumption of homogeneity of variance was violated, and the data were negatively skewed, violating the assumption of normal distribution. Transformations of the data were attempted, but this did not reduce the skew. A number of cases appeared as outliers for all outcome variables, and notably, these cases were all from the housekeeping department. Data were therefore examined using non-parametric methods. This point was discussed with our statistician, who assured us that the loss of power associated with the non-parametric tests was small.

Therefore, internal consistency was examined using Spearman's Rho correlation to calculate the Corrected Item-Total Correlation (CITC) coefficient values for subscales of the STA and the RSE. Convergent validity was analysed by correlating subscales of the STA scale and the RSE using Spearman's Rho. The Sidak adjustment was used to adjust for multiple comparisons.

Non-parametric statistical tests were used to examine the association between scores on the STA and the RSE, and the variables requested in the demographic questionnaire (length of experience working in secure services, the ward and level of security worked on, the gender of the patients on their ward, and staff department/occupational discipline). Analysis between individual wards and staff occupational discipline could not be completed due to small and unequal numbers between the groups. To examine the association between level of security and the subscales of the two measures, the Kruskal-Wallis test was used. The Wilcoxon Mann-Whitney *U*-test was used to examine the association between gender of patients and the subscales. To examine the association between length of experience working in secure services and the subscales, Spearman's Rho correlation was used.

## Results

### Internal consistency

Internal consistency was assessed using CITC coefficients. A CITC value above 0.5 is considered high, but if less than 0.3, items within a subscale may be measuring more than one construct. All RSE subscales exceeded the CITC 0.30 cut-off, although there was some variation, with the Personal World subscale having the highest internal consistency at 0.80, and the Physical Environment subscale the lowest at 0.49. All the STA subscales had CITC scores over 0.9. Table 1 displays the CITC coefficient values for each subscale of the two measures.

**Table 1:** Corrected Item-Total Correlation (CITC)

Measure	CITC
Relational Security Explorer	
Therapy	0.66
Boundaries	0.61
Patient Mix	0.57
Patient Dynamic	0.65
Personal World	0.80
Physical Environment	0.49
Visitors	0.62
Outward Connections	0.68

See Think Act scale	
Therapeutic Risk Management	0.90
Pro-Social Team Culture	0.96
Boundaries	0.92
Patient Focus	0.92

### Convergent validity between measures

There was little convergent validity between the subscales of the two measures. All four subscales of the STA scale correlated significantly with each other, whereas subscales of the RSE did not. The Spearman's Rho values are detailed in Table 2.

**Table 2:** Correlations matrix of the subscales of See Think Act scale and Relational Security Explorer

	See Think Act scale				Relational Security Explorer							
Measure	Therapeutic Risk Management	Pro Social Team Culture	Boundaries	Patient Focus	Therapy	Boundaries	Patient Mix	Patient Dynamic	Personal World	Physical Environment	Outward Connections	Visitors
See Think Act scale												
Therapeutic Risk Management	1											
Pro Social Team Culture	0.808**	1										
Boundaries	0.780**	0.878**	1									
Patient Focus	0.812**	0.846**	0.795**	1								

Relational Security Explorer												
Therapy	0.424	0.436	0.344	0.413	1							
Boundaries	0.470	0.411	0.367	0.341	0.469**	1						
Patient Mix	0.444	0.468	0.582**	0.401	0.338	0.293	1					
Patient Dynamic	0.453	0.482	0.457	0.401	0.515**	0.300	0.668**	1				
Personal World	0.485	0.487	0.391	0.465	0.588**	0.439**	0.319	0.447**	1			
Physical Environment	0.091	0.182	0.247	0.246	0.251	0.249	0.110	0.173	0.239	1		
Outward Connections	0.320	0.236	0.171	0.278	0.273	0.266	0.297	0.300	0.556**	0.186	1	
Visitors	0.301	0.262	0.211	0.247	0.207	0.237	0.267	0.235	0.573**	0.145	0.565**	1

** Correlation is significant at the 0.01 level.

### Between-groups analysis

#### Level of security

Significant differences in scores on the two measures were found between staff working in different levels of security Staff working on medium secure wards tended to have the lowest scores on both measures, followed by staff on low secure wards, with the highest scores on rehabilitation wards. On the STA scale, this reached statistical significance on the Therapeutic Risk Management (*P*< 0.001), Boundaries (*P* = 0.012) and Patient Focus (*P* = 0.034) subscales. There were also significant differences on the RSE, on the Patient Mix (*P*< 0.001), Patient Dynamic (*P* = 0.008) and Personal World (*P* = 0.011) subscales. Descriptive data for this variable are presented in Table 3.

**Table 3:** Descriptive statistics for level of security on the See Think Act scale and Relational Security Explorer

	Level of security, mean (s.d.)		
Measure	Medium *n* = 13	Low *n* = 17	Rehabilitation *n* = 14
See Think Act scale^a^			
Therapeutic Risk Management	2.34 (0.44)	2.54 (0.39)	2.8 (0.25)
Pro-Social Team Culture	2.34 (0.49)	2.52 (0.48)	2.68 (0.41)
Boundaries	2.5 (0.43)	2.64 (0.41)	2.83 (0.31)
Patient Focus	2.5 (0.45)	2.76 (0.49)	2.76 (0.34)

Relational Security Explorer^b^			
Therapy	7.46 (1.61)	7.47 (0.26)	8.07 (1.61)
Boundaries	7.85 (1.21)	8.3 (0.26)	8.3 (1.21)
Patient Mix	7.54 (0.97)	7.1 (1.56)	8.71 (0.61)
Patient Dynamic	7.15 (1.34)	7.1 (1.34)	8.3 (0.83)
Personal World	7.31 (1.7)	7.41 (1.18)	8.5 (1.09)
Physical Environment	7.23 (1.7)	7.88 (1.4)	8.07 (1.33)
Visitors	7.0 (1.09)	7.0 (1.12)	7.64 (1.98)
Outward Connections	7.23 (1.36)	7.17 (1.74)	8.07 (1.59)

a. The scale for the See Think Act scale is 0–3.

b. The scale for the Relational Security Explorer is 1–9.

#### Ward gender

There were no differences in STA scale scores between staff working on wards caring for male patients, as compared to female patients. However, on the RSE, staff working with male patients reported higher scores on the Patient Dynamic (*P* = 0.024) subscale, compared with staff working with female patients.

#### Length of experience working in secure services

There was no correlation between the number of years staff had been working in secure services, and their confidence in relational security, on the STA scale or the RSE.

## Discussion

Serious incidents within forensic mental health services can be linked to breakdowns of relational security.^1^ Available measures should therefore provide insight into the quality of relational security within services, direct exploration of strengths and weaknesses, and prompt timely, appropriate interventions before an incident occurs. Furthermore, tools should be accessible to all occupation groups.^6^ This study therefore evaluated the psychometric properties of the STA and the RSE, including internal consistency, convergent validity and their ability to highlight differences between groups.

Study limitations include a relatively small sample size, and participants being drawn from a single service, which limit generalisability. It is categorised as a preliminary study for these reasons. However, the study reports interesting findings in relation to the specific tools examined and the wider task of measuring relational security by investigating the perspectives of staff members across occupational professions and levels of security. The study setting, a secure intellectual disability service, may be viewed as a strength, as previous studies have been completed only within generic forensic psychiatric services, and as a weakness, due to the extent of the study findings generalising to non-intellectual disability services. Research comparing patient characteristics between mainstream forensic and specialist intellectual disability services is scarce, however there are a number of notable differences between the two populations which may have an effect on relational security. These include communication difficulties which could affect the development of therapeutic relationships, increased levels of behavioural incidents,^12^ and higher assessed risk on structured clinical judgement tools.^13–15^ That said, the present research reports many findings which echo those of previous research.^2^

Test–re-test reliability was not examined in the present research, although it may be an interesting area for further study. At present, the stability of relational security levels are unclear, and it may not be realistic or relevant to expect stability over time, as relational security is a fundamentally a dynamic concept.

The internal consistency aspect of reliability was examined, with the STA scale demonstrating high levels in accordance with previous research.^2^ Its subscales correlated significantly, suggesting they are statistically related concepts. Internal consistency for the RSE was adequate but its subscales did not all correlate significantly, possibly indicating that some of the concepts measured are statistically unrelated. Although the RSE and the STA both aim to measure relational security, convergent validity was low. Collectively, these findings suggest that the RSE does not perform as well as the STA as a psychometric outcome measure. This raises questions regarding the practice of collecting and analysing data from the RSE, despite not being validated for this purpose. Lodewijks *et al*^9^ explicitly advise against the use of numerical indices and cut-off points, due to clinicians' tendency to reduce clinical decisions to numbers if they are available.

Particular subscales of the two measures were able to detect differences between groups in relational security confidence, thus facilitating the comparison of wards and staff disciplines within a service, potentially between services comparisons, and directing intervention as necessary. Significant findings were reported between wards of different levels of security, with relational security confidence lowest on medium secure wards, increasing on low secure wards, and highest on rehabilitation wards. Tighe & Gudjonsson^2^ also reported this effect, as well as authors investigating social climate in secure settings.^16,17^ A possible explanation for this is that medium secure wards represent the beginning of the care pathway accepting newly admitted patients and, as such, staff have had less opportunity to develop the knowledge of patients and therapeutic relationships necessary to achieve good relational security. As patients progress through the low secure and rehabilitation stages of the care pathway, this provides an opportunity for the components necessary to relational security to develop.

The study also compared relational security confidence between staff working with male and female patients. This was examined as authors have documented difficulties in building therapeutic relationships with women, due to their clinical complexity.^18,19^ However, there were few differences between these two groups on the subscales of the two measures, although staff working with male patients reported higher scores on the Patient Dynamic (*P* = 0.024) subscale of the RSE.^5^

It was expected that staff with more experience working within secure services would be more confident in their judgements of relational security, but there were no significant associations. However, relational security relies on knowledge of patients and therapeutic relationships, within a dynamic environment. For example, if a new patient is admitted to a ward, all staff, regardless of experience will begin to develop their knowledge and their therapeutic relationships with this patient at the same time. Furthermore, if an experienced staff member is asked to move onto a different ward, they will know little about the patients on that ward and have to develop new knowledge and therapeutic relationships. In this sense, all staff members, regardless of experience, are repeatedly beginning and developing their knowledge of individual patients and new therapeutic relationships.

Housekeeping staff felt less confident in relational security, as compared to all other departments. This is probably due to the measures tapping into areas of clinical practice that housekeeping staff would not be involved in, and the comparable lack of training offered to this group. However, guidance recommends that relational security implementation should involve all occupational groups working within secure services.^6^ As housekeeping staff are a daily presence on forensic wards, they are equally as vulnerable to relational security issues and in a position to witness threats to security. Further research should investigate ways to support all occupational groups with relational security.

### Measuring relational security: further considerations

Some points are of note when interpreting data obtained from relational security measures. Initial assumption testing indicated negatively skewed data, suggesting that most participants rated their confidence in relational security at the top end of each measures' respective scale. This could indicate that staff working in this particular service are highly confident in their relational security practice. However, it is unclear how confidence scores relate to the actual quality of relational security within a ward, for example is a highly confident team an experienced team or a complacent team? It could also indicate positive responding, as essentially, measures are asking individuals to rate themselves in an aspect of their role in which they are expected to be competent. Introducing some negatively worded items could improve this.

High scoring may also be due to the way the two tools conceptualise relational security. Both measures are based on the STA conceptualisation of relational security,^6^ which places much of the responsibility onto staff and teams, thus neglecting the ‘quantitative’ aspects of relational security, for example ‘staff-to-patient ratio and amount of time spent in face-to-face contact’ (p. 434)^20^ and the supportive role and responsibility of service management. For example, to achieve quality therapeutic relationships with patients, ward staff and teams need to be stable, with minimum staff ward moves, turnover and absenteeism,^3^ factors outside of staff members' control. Incorporating items reflecting such aspects of relational security could provide a more comprehensive picture.

Normative data are not currently available for either of the two measures, which limits the interpretability of the results. Both measures are designed to be used by staff members in relation to one specific ward. However, many employees work across multiple wards in secure services, with 27% respondents in this sample working across wards. These participants were typically from occupational departments other than nursing, such as psychology, psychiatry, social work and occupational therapy.

### Conclusions

The increased attention relational security is receiving within the forensic field is a welcome advance. There is growing awareness around the importance of this concept, and resources aiming to raise awareness and support the implementation of relational security are widely available.^21^ However, until recently there have been few mechanisms to assess the quality of relational security within services. The results of this study suggest that the STA scale and the RSE hold unique and complimentary roles attempting to support relational security. The RSE did not perform well as a psychometric measure, and therefore its use should be limited to its intended purpose – as a tool to guide team discussions about relational security – and services should refrain from using the RSE as an outcomes measure. However, deciding on a numerical score of relational security confidence may serve a function within the context of a team discussion, for example, if one team member feels the ward should score highly, whereas another member feels the ward should be given a lower score, this could suggest team splitting and form a basis for discussion. At present, the STA scale is best placed to provide insight into the quality of their relational security, while taking into account the aforementioned issues when interpreting the data.

Further research is needed in all areas of relational security: definition, implementation and measurement. This research should examine the relationship between relational security and negative outcomes, such as institutional aggression or serious incidents. Future studies should evaluate the clinical utility of the RSE, examine the psychometric properties and provide normative data for the STA. Until more is known about relational security, the approach to implementing and measuring it should be multidimensional,^22^ i.e. incorporating staff, patient and service management perspectives.

## References

[1] Allen E See Think Act: Relational Security in Secure Mental Health Services. Department of Health, 2010.

[2] Tighe J Gudjonsson GH See, Think, Act Scale: preliminary development and validation of a measure of relational security in medium- and low-secure units. J Forens Psychiatry Psychol 2012; 23: 184–99.

[3] Chester V Morgan W Relational security within secure services: summary of findings from a literature review. Quality Network for Forensic Mental Health Services Newsletter 2012; 9: 10–4.

[4] Dale C Storey L High, medium and low security care: does the type of care make any difference to the role of the forensic mental health nurse? Res Nurs 2004; 9: 168–84.

[5] Department of Health Relational Security Explorer. Department of Health, 2010.

[6] Department of Health Your Guide to Relational Security: See, Think, Act. Department of Health, 2010.

[7] Johnson S Wood S Paul M Osborn D Wearn E Lloyd-Evans B Inpatient Mental Health Staff Morale: A National Investigation. National Institute for Health Research, 2011.

[8] Schalast N Redies M Collins M Stacey J Howells K EssenCES, a short questionnaire for assessing the social climate of forensic psychiatric wards. Crim Behav Ment Health 2008; 18: 49–58. 18229876 10.1002/cbm.677

[9] Lodewijks HPB Doreleijers TAH De Ruiter C Savry risk assessment in violent dutch adolescents: relation to sentencing and recidivism. Crim Justice Behav 2008; 35: 696–709.

[10] NHS Health Research Authority Defining Research. NHS Health Research Authority, 2016.

[11] Department of Health Research Governance Framework for Health and Social Care. NHS Health Service Authority, 2005.

[12] NHS Benchmarking Network Use of Restraint in Mental Health, CAMHS and LD Phase 2 Data Collection: Participant Feedback. 2015.

[13] Alexander RT Chester V Gray NS Snowden RJ Patients with personality disorders and intellectual disability – closer to personality disorders or intellectual disability? A three-way comparison. J Forens Psychiatry Psychol 2012; 23: 435–51.

[14] Morrissey C Beeley C Milton J Longitudinal HCR-20 scores in a high-secure psychiatric hospital. Crim Behav Ment Heal 2014; 24: 169–80. 10.1002/cbm.1893 24265120

[15] Fitzgerald S Gray NS Alexander RT Bagshaw R Chesterman P Huckle P Predicting institutional violence in offenders with intellectual disabilities: the predictive efficacy of the VRAG and the HCR-20. J Appl Res Intellect Disabil 2013; 26: 384–93. 23925961 10.1111/jar.12032

[16] Quinn M Thomas C Chester V The Essen Climate Evaluation Schema measure of social climate in a secure service for people with intellectual disabilities. Adv Ment Heal Intellect Disabil 2012; 6: 171–8.

[17] Langdon PE Swift A Budd R Social climate within secure inpatient services for people with intellectual disabilities. J Intellect Disabil Res 2006; 50(Pt 11): 828–36. 16999782 10.1111/j.1365-2788.2006.00847.x

[18] Aiyegbusi A Thinking under fire—the challenge for forensic mental health nurses working with women in secure care. In Working Therapeutically with Women in Secure Mental Health Settings (ed Jeffcote N): 108–19. Jessica Kingsley, 2004.

[19] Chester V Alexander RT Women with intellectual disabilities and forensic involvement. In The Wiley Handbook on Offenders with Intellectual and Developmental Disabilities (eds Lindsay WR Taylor JL). John Wiley and Sons, in press.

[20] Kennedy HG Therapeutic uses of security: mapping forensic mental health services by stratifying risk. Adv Psychiatr Treat 2002; 8: 433–43.

[21] Allen E Your Guide to Relational Security: See Think Act (2nd edn). Royal College of Psychiatrists Quality Network for Forensic Mental Health Services, 2016.

[22] Parry-Crooke G Stafford P My Life: In Safe Hands? Dedicated Women's Medium Secure Services in England. London Metropolitan University, 2009.

